# Clinical outcomes of gastrointestinal brain metastases treated with radiotherapy

**DOI:** 10.1186/s13014-017-0774-3

**Published:** 2017-02-28

**Authors:** Samrat M. Sanghvi, Jonathan W. Lischalk, Ling Cai, Sean Collins, Mani Nair, Brain Collins, Keith Unger

**Affiliations:** 1 Georgetown University School of Medicine, Medical Dental Building, 3900 Reservoir Road, N.W, Washington DC, 20057 USA; 2 Department of Radiation Medicine, Lombardi Comprehensive Cancer Center, Georgetown University Hospital, Lower Level Bles, 3800 Reservoir Road, N.W, Washington DC, 20007 USA; 3 Department of Biostatistics, Bioinformatics, and Biomathematics, Georgetown University, 4000 Reservoir Rd., NW, Washington DC, 20057 USA; 4 Department of Neurosurgery, 3800 Reservoir Rd. NW, Pasquerilla Healthcare Center Seventh Floor, Washington DC, 20007 USA

**Keywords:** Radiosurgery, Gastrointestinal neoplasms, Brain neoplasm, Metastases

## Abstract

**Background:**

Brain metastases of gastrointestinal origin are a rare occurrence. Radiation therapy (RT) in the form of stereotactic radiosurgery (SRS) or whole brain radiation therapy (WBRT) is an effective established treatment modality in either the definitive or adjuvant setting. The aim of this study is to assess the long-term clinical outcomes of patients with gastrointestinal (GI) brain metastases treated with SRS or WBRT.

**Methods:**

In this single institutional retrospective review, we detail the outcomes of patients diagnosed with metastatic brain tumors from an adenocarcinoma gastrointestinal primary. Patients were treated using stereotactic radiosurgery or whole brain radiation therapy. Initial site control (defined as lesions visualized on imaging at time of treatment), new site control (defined as new intracranial lesions visualized on follow-up imaging), and overall survival were calculated using the Kaplan-Meier method.

**Results:**

Thirty-three patients were treated from August 2008 to December 2015. Primary malignancy locations were as follows: 18 colon, 6 esophagus, 4 rectum, 5 other. Median total dose delivered was 25 Gy (18–35 Gy) in a median of 4 fractions for SRS and 30 Gy (10.8–40 Gy) in 10 fractions for WBRT. Crude initial site control at last radiographic follow-up was 64.3% after SRS and 41.7% after WBRT. Eleven of the 28 brain lesions (39.3%) treated with SRS had resection of the SRS-treated lesion prior to radiation therapy. Five of the twelve patients (41.7%) undergoing WBRT underwent cranial resection prior to radiation therapy. Crude new site control at last radiographic follow-up was 46.4% after SRS and 83.3% after WBRT. Kaplan-Meier analysis of overall survival did not show any statistically significant difference between WBRT and SRS (*p* = 0.424). Median overall survival for SRS patients was 5.2 months (0.5–57.5) and for WBRT patients 4.4 months (0–15). Kaplan-Meier analysis of new site control was significantly improved with WBRT versus SRS (*p* = 0.017). Total dose, treatment with WBRT, and active extracranial disease were statistically significant on multivariate analysis for new site control (*p* < 0.05).

**Conclusions:**

Survival and intracranial disease control are poor following RT for brain metastases from GI primaries. In this small series, outcomes are worse than published series for other primary malignancies metastatic to the brain and further research into methods of local control improvement is warranted. Future studies should explore the utility of dose escalation or radiosensitization in this patient population.

## Introduction

Gastrointestinal (GI) malignancies represent a significant burden of disease in the United States with colorectal cancer representing nearly 10% of all new cancer diagnoses and ranking as the second leading cause of cancer related death [1]. These sites rarely metastasize to the brain with reported rates at less than 9% [2–6]. Nevertheless, given the prevalence of GI malignancies, brain metastases from these primaries still represent 4–8% of all diagnosed brain metastases [3, 7–9]. Moreover, their incidence is increasing possibly due to longer survival seen in patients with colorectal primaries as a result of improvements in systemic therapy and earlier diagnosis [10, 11]. Additionally, wider utilization of brain MRIs has contributed to an increased diagnosis of previously occult brain metastases [12].

Historically, whole brain radiation therapy (WBRT) has been the standard of care for brain metastases given the lack of accurate targeting coupled with the concern for microscopic disease elsewhere in the brain [13]. With improving targeting technology however, stereotactic radiosurgery (SRS) has emerged as an alternative treatment modality for many cases of metastatic disease to the brain. As compared to WBRT, SRS has the potential to be completed in less time, achieve increased efficacy against radioresistant tumors, increase the likelihood of re-irradiation, decrease hair loss, and mitigate neurocognitive side effects [14–17].

Literature documenting clinical outcomes of patients with brain metastasis from GI primaries treated with radiation therapy is scarce and focuses mainly on outcomes following Gamma Knife radiosurgery (GKS) and not LINAC-based techniques, which are becoming a more prevalent modality of SRS. The purpose of this retrospective review is to explore the clinical outcomes, local and distant intracranial control rates, and prognostic factors associated with GI brain metastasis treated with WBRT and SRS.

## Materials and methods

### Patient eligibility

The local Institutional Review Board approved this retrospective analysis of an established departmental treatment approach. The analysis was conducted utilizing the medical records of patients diagnosed with a metastatic brain tumor from a primary gastrointestinal malignancy between 2008 and 2015. Inclusion criteria was as follows: (1) primary adenocarcinoma malignancy originating from the colon, rectum, pancreas, esophagus, rectosigmoid, duodenum, or stomach, (2) histologic confirmation of primary gastrointestinal malignancy, and (3) brain metastasis confirmed by computed tomography (CT) scan or by magnetic resonance imaging (MRI). Exclusion criteria were as follows: (1) prior history of malignancy within the last 5 years, and (2) lack of radiographic follow-up (specifically for intracranial control analyses). Patients without radiographic follow-up were included in the overall survival analysis. Lesions that underwent surgical resection prior to radiotherapy were included. Sites of distant extracranial disease were identified utilizing positron emission tomography (PET), skeletal scintigraphy, CT, and/or MR imaging as deemed necessary by medical oncology performed within 2 months of original intracranial metastatic diagnosis. If no imaging modality was available within 2 months, distant extracranial disease was determined by clinical exam of the primary medical oncologist in conjunction with prior imaging at the time of development of brain metastases. Twelve patients had PET scans for extracranial disease determination, fifteen utilized a CT of the thorax, abdomen, and pelvis with contrast in conjunction with prior PET scans, five utilized alternate imaging modalities in conjunction with older imaging, and one patient was staged clinically with utilization of alternate imaging modalities. Extracranial disease status was determined by the official radiology report [18].

### Treatment planning and delivery

Patients were treated in the supine position with a custom aquaplast mask for reproducible patient set-up. A fine-cut contrast enhanced treatment planning CT scan of the brain was obtained for each patient in the supine treatment position using a GE LightSpeed RT16. SRS treatment planning and delivery was done on the CyberKnife Robotic SRS system as detailed previously [19]. WBRT was performed with 6× photons delivered using opposed laterals with custom MLC blocking and a thermoplastic mask. The radiation therapy modality (SRS vs. WBRT) was selected at the discretion of the treating radiation oncologist, based on the number of lesions, lesion size, location of the lesions, and the patient’s performance status. The radiation dose for WBRT and SRS was selected at the discretion of the treating radiation oncologist; SRS dose and fractionation was primarily based on target volume and location within or adjacent to an eloquent area of the cortex.

### Follow up

Patients were followed with MRI or CT imaging at three to six month intervals according to routine institutional practice. Initial site control was defined as no progression in size of the initial tumor. Progression was defined based on increased tumor size, T1 post contrast enhancement, mass effect, fluid-attenuated inversion recovery, and/or vasogenic edema. For WBRT, initial site was defined as all tumors seen on brain imaging at time of treatment, and for SRS was specified as the treated tumor. New intracranial lesions were defined as new brain masses of any size noted on follow-up imaging. New site control was defined as no new development of intracranial lesions. If new intracranial lesions were noted, this was defined as a failure of new site control. We were unable to determine the cause of death from the available medical records in a sufficient number of patients.

### Statistical analysis

Statistical analysis was performed with the R statistical software, version 3.2.1 (R Foundation for Statistical Computing, http://www.r-project.org, Vienna, Austria). Baseline patient and tumor values (age, treatment length, dose, etc.) were analyzed for descriptive characteristics (mean, median, etc.). The follow-up duration was defined as the time from treatment completion to the last date of imaging follow-up. Tumors were censored at the date of last radiographic follow-up for initial and new site control analysis. Kaplan-Meier estimates were used for the calculation of overall survival (OS), initial site control, and new site control. The log-rank test and the Cox proportional hazards model were used to analyze the factors affecting overall survival, initial site control, and new site control. A *p* value < 0.05 was regarded as statistically significant.

## Results

### Patient characteristics

Thirty-three patients were treated from August 2008 to December 2015. The median age was 62 years (29 to 83 years). The majority of patients were male (66%) and median pretreatment ECOG performance status was 1 (0 to 3). Distribution of primary tumor location was as follows: 18 colon, 6 esophagus, 4 rectum, 2 gastric, 1 rectosigmoid, 1 duodenal, and 1 pancreas. The majority of patients had active extracranial disease at the time of diagnosis of brain metastases (87.9%). Twenty patients had liver metastases (60.6%) and 20 patients had lung metastases (60.6%), with 16 of those patients having both liver and lung metastases (48.5%) at the time of presentation with brain metastases. Median number of brain tumors at initial presentation was two (1 to 5). Summary patient characteristics are shown in Table 1. Two patients had brain metastases at initial primary site cancer diagnosis. Median time between initial cancer diagnosis and brain metastasis diagnosis was 18.1 months. Of those with radiographic follow-up, 12 patients were treated with WBRT and 16 patients were treated with SRS. Eleven of the 28 brain lesions (39.3%) treated with SRS had resection of the SRS-treated lesion prior to radiation therapy. Five of the twelve patients (41.7%) undergoing WBRT underwent cranial resection prior to radiation therapy. Median time between surgery and radiation therapy was 28 days with a mean of 46.33 days. One lesion was treated with SRS followed by planned WBRT.

**Table 1:** Patient characteristics

Treatment				
	WBRT	SRS	Total	
	(*n* = 12)	(*n* = 21)	(*n* = 33)	*P*-value
Median age (range)	62 (41 ~ 76)	62 (29 ~ 83)	62 (29 ~ 83)	0.963
Median interval between cancer diagnosis and brain metastases (range)	422.5 (0 ~ 2155)	905 (22 ~ 1668)	542 (0 ~ 2155)	0.779
Brain metastases at initial presentation [*N* (%)]				
Yes	2 (100)	0 (0)	2	0.125
No	10 (32)	21 (68)	31	
Intracranial surgery prior to radiation therapy [*N* (%)]				
No	7 (50)	7 (50)	14	0.273
Yes	5 (26)	14 (74)	19	
Primary site [*n* (%)]				
Colon	4 (24)	13 (76)	17	0.247
Esophagus	3 (50)	3 (50)	6	
Other	4 (67)	2 (33)	6	
Rectum	1 (25)	3 (75)	4	
BMs [*n* (%)]				
≤2	4 (17)	19 (83)	23	0.001*
>2	8 (80)	2 (20)	10	
ECOG [*N* (%)]				
Unknown	0 (0)	1 (100)	1	0.273
≤1	8 (50)	8 (50)	16	
>1	4 (25)	12 (75)	16	
RPA class [*N* (%)]				
Unknown	0 (0)	1 (100)	1	0.144
I and II	8 (53)	7 (47)	15	
III	4 (24)	13 (76)	17	

*Abbreviations*: *WBRT* whole brain radiation therapy, *SRS* stereotactic radiosurgery, *BMs* brain metastases, *ECOG* Eastern Cooperative Oncology Group, *RPA* recursive partitioning analysis

Statistically significant results indicated by *

### Treatment characteristics

Table 2 shows baseline tumor and treatment characteristics. Median total dose delivered was 25 Gy (18–35 Gy) for SRS and 30 Gy (10.8–40 Gy) for WBRT. Median number of fractions for SRS was four (1–4) and for WBRT was ten (6–20). Median gross tumor volume (GTV) treated with SRS was 4.6 cc (0.11–95.19 cc) and mean GTV was 12.08 cc. Biologically equivalent dose (BED) was calculated utilizing the linear-quadratic approach with an alpha/beta value of ten. For SRS the median BED was 48 Gy (37.5 to 93.6 Gy), and for WBRT it was 39 Gy (12.74–48 Gy).

**Table 2:** Tumor and treatment characteristics

	SRS (*n* = 28)	WBRT (*n* = 12)
Median tumor volume in cubic centimeters (range)	4.6 (0.11–95.19)	–
Median tumor coverage (range)	99.41 (97.9–100)	–
Median Dose per fraction in cGy (range)	750 (500–2600)	300 (180–300)
Median fractions (range)	4 (1–5)	10 (6–20)
Median total dose in cGy (range)	2500 (1800–3500)	3000 (1080–4000)
Median biologically equivalent dose in Gy (range)	48 (37.5–93.6)	39 (12.74–48)
Median length of treatment in days (range)	4 (1–12)	15 (14–71)
Prior WBRT	2	0

*Abbreviations*: *SRS* stereotactic radiosurgery, *WBRT* whole brain radiation therapy, *Gy* gray

**Fig. 1:**
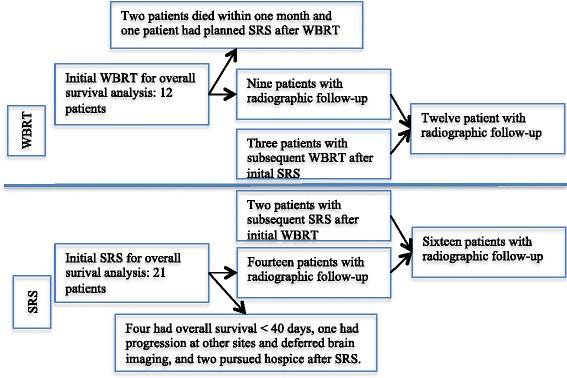
Patient attrition

Overall, five patients received SRS salvage after initial WBRT and six patients received WBRT salvage after initial SRS. The following lesions were excluded from the new site control analysis for the SRS group: (1) one patient with planned WBRT after SRS, and (2) two patients with previous WBRT within 6 months. Figure 1 shows patient attrition through the study. The radiographic follow-up for the initial treated lesion was one scan for ten patients, two scans for five patients, three scans for four patients, and equal to or greater than four scans for four patients.

### Outcomes

**Fig. 2:**
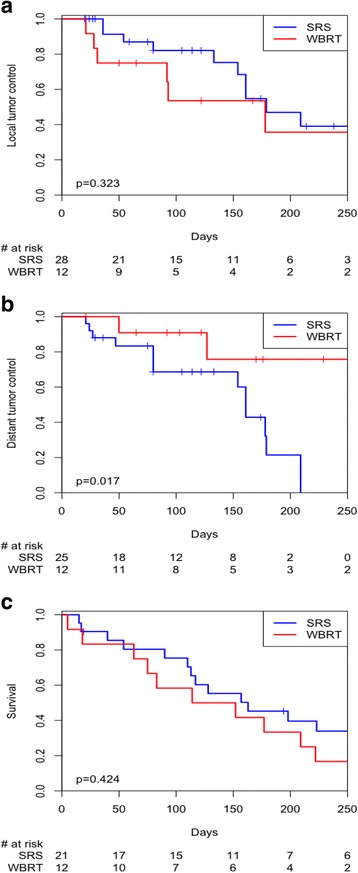
**a** Kaplan-Meier initial lesion control. **b** Kaplan-Meier new intracranial lesion control (also referred to as distant intracranial control). **c** Kaplan-Meier overall survival

Crude initial site control (also referred to as local control for SRS) at last radiographic follow-up was 64.3% after SRS and 41.7% after WBRT. Crude new site control (also referred to as distant intracranial control for SRS) defined as no new lesions seen within the brain at last radiographic follow-up were 46.4% after SRS and 83.3% after WBRT. Median radiographic follow-up for SRS was 3.9 months (0.7–57.3) and for WBRT was 4.1 months (0.7–53.8). Kaplan-Meier analysis for initial site control and new site control is shown in Fig. 2a and b respectively. Kaplan-Meier analysis for overall survival is shown in Fig. 2c. For SRS, the actuarial 6-month local control was 47%, the actuarial 6-month new intracranial lesion control was 21%, and the actuarial 6-month overall survival was 45%. Median overall survival for SRS patients was 5.2 months (0.5–57.5) and for WBRT patients 4.4 months (0–15). There was no statistically significant difference between the two treatments in regards to initial site control and overall survival as determined by Kaplan-Meier analysis (*p* = 0.323 and 0.424, respectively). There was a significant difference between the two treatments in regards to new site control, which was found to be superior in the WBRT group (*p* = 0.017).

In univariate analysis (UVA) for overall survival, the following variables were analyzed: age, total dose, treatment length, interval between initial cancer diagnosis and brain metastasis diagnosis, surgery prior to RT for largest lesion, presence of extracranial disease, number of brain metastases (≤2 vs >2), ECOG (≤1 vs >1), RPA class, and site of primary disease. None of these variables were statistically significant (results not shown). In addition, none of the variables analyzed on multivariate analysis (MVA) of overall survival were found to be statistically significant (results shown in Table 3).

**Table 3:** Multivariate analysis for overall survival, initial site control and new site control

Characteristic	HR (95% CI)	*P*-value
Overall survival		
Age	0.99 (0.95–1.02)	0.451
ECOG	1.63 (0.69–3.86)	0.263
>2 brain metastases	0.53 (0.12–2.25)	0.388
Active extracranial disease	3.02 (0.62–14.79)	0.173
Intracranial Surgery prior to RT	0.62 (0.24–1.61)	0.326
Treatment WBRT	1.39 (0.45–4.22)	0.566
Initial site control		
Total Dose	0.69 (0.31–1.55)	0.372
Treatment WBRT	1.95 (0.68–5.6)	0.212
Intracranial Surgery prior to RT	0.47 (0.16–1.36)	0.164
New site control		
Total Dose	3.98 (1.13–14.07)	0.032*
Treatment WBRT	0.05 (0.01–0.35)	0.002*
Active extracranial disease	11.4 (1.54–84.52)	0.017*

*Abbreviations*: *CI* confidence interval, *HR* hazard ratio, *ECOG* Eastern Cooperative Oncology group

Statistically significant results indicated by *

**Table 4:** Published series of local control for patients with brain metastases from gastrointestinal primaries

Study	Year	Number of patients	Number of tumors	Treatment	Previous cranial RT	LC at last follow-up	Median radiographic follow-up	Median OS in months	Primary malignancy	Local Progression definition	Tumor volume	Adeno- carcinoma
Trifiletti et al. [13]	2015	86		GKS	19.80%	94%	4.6 months (mean 7.7 months)	6.2	GI	Tumors with a volume increase of 10%	mean 3.7 (0.7 ml median)	59.30%
Matsunaga et al. [24]	2011	152	616	GKS	6%	91%	3.0 months (mean 6.4 months)	6	Colorectal	Tumors with an increase greater than 25% in maximum diameter	mean 2.0 cm3 (0.004–10.0 cm3)	“almost all”
Da Silva et al. [9]	2009	40	118	GKS	47.50%	91%	–	6.7	GI	Tumors with a volume increase of > 15%	mean 4.3 cm3 (0.01–35)	–
Current Series	2016	23	28	CK	7.14%	64.30%	3.9 months (mean 6.3 months)	5.2	GI	Increase in tumor size as determined by neuroradiologist	mean 12.1 cm3 (0.2–95.1 cm3)	100%

*Abbreviations*: *RT* radiation therapy, *OS* overall survival, *GKS* Gamma Knife Surgery, *GI* gastrointestinal

In UVA for initial site control and new intracranial lesion control, the following variables were analyzed: surgical resection prior to RT, number of brain metastases, total dose, tumor volume, and tumor coverage. None of these variables were found to be statistically significant (results not shown). On MVA for initial site control, none of the analyzed variables were found to be statistically significant (results shown in Table 3). On MVA for new site control, the following variables were found to be statistically significant: total dose, treatment with WBRT, and having active extra cranial disease (results displayed in Table 3).

## Discussion

Brain metastases are generally considered a late event for patients with gastrointestinal primaries with reported rates at presentation of only 0.2% [5]. Hence, patients rarely present with only metastases to the brain and more commonly develop liver or lung metastases prior to the development of metastatic brain foci [5]. This is consistent with what was found in this series with 72.7% of patients presenting with liver and/or lung metastases at the time of brain metastases diagnosis. Though rare, brain metastases are an often fatal and morbid aspect of disease progression, and radiation therapy has become an accepted treatment modality for these metastatic lesions [9, 10]. However, there is also a high competing risk of death from the significant burden of extracranial disease present in this patient population.

SRS for GI brain metastases has been documented to provide local tumor control rates of 84 to 96% [9, 10, 20–22]. Our results show a crude local control rate of 64.3%. This is much lower than other reported rates, which may be explained by differences in prior radiation therapy, patient population, treatment modality, radiation doses, percentage adenocarcinoma histology, lesion sizes, and local failure definitions. Many studies of the clinical outcomes for GI brain metastases have included patients who previously received RT [9, 10, 22]. For instance, Da Silva et al. included 55% of patients who had previously received cranial RT, and Trifiletti et al. included 36.1% of patients [9, 10]. In contrast, Matsunaga et al. included only 5.9% of patients having previously received WBRT similar to our study which included only two lesions (7.14%) with WBRT prior to SRS [22]. Matsunaga et al. found a crude local control rate of 91%, however they defined local failure as an increase greater than 25% in maximum diameter and had a shorter median radiographic follow-up than the current series [22]. Additionally, our series treated larger lesions (mean size of 12.1 cc) than the previous studies [9, 10, 22]. Larger colorectal brain lesions have been shown to have worse local control following RT [22]. Furthermore, other studies have treated a smaller portion of adenocarcinoma histology than our current series, which treated exclusively adenocarcinoma [9, 10, 22]. Table 4 summarizes previous studies that have reported crude local control rates after RT for GI brain metastases.

The overall median survival found in this study was 5.1 months and previous studies have shown similar findings with poor outcomes following the development of GI brain metastases. A study performed by Ogawa et al. and Weinberg et al. showed a median OS of 6.7 months and 2.3 months, respectively, after the development of brain metastases from esophageal carcinoma. York et al. reported a 2.3 month median OS from gastric cancer brain metastases, and both Schoeggl et al. and Matsunaga et al. reported median survival of 6.0 months after colorectal brain metastases. This poor overall survival is worse than other published data from non-GI primaries and is indicative of the importance in identifying prognostic factors associated with these poor clinical outcomes to appropriately allocate dose escalation and/or concurrent use of radiosensitizers [23]. Furthermore, identifying prognostic factors for poor overall survival can help us better advise patients regarding hospice management and palliative care.

Other studies analyzing GI brain metastases have found improved local control with margin dose ≥ 20 Gy and improved survival in luminal primaries (esophagus, small bowel, stomach, colon and rectum) versus non-luminal primaries (pancreas and liver) [10]. A study analyzing esophageal carcinoma brain metastases found that a higher KPS was associated with an increase in survival [24]. Furthermore, a study analyzing colorectal brain metastases found unfavorable local control with larger tumor volume and lower margin dose, while poor OS was associated with lower KPS and the presence of extracranial metastases [22]. Our study did not identify significant differences on UVA and MVA in the variables analyzed for overall survival and local control. Prior studies have demonstrated improved local control with SRS as compared to whole brain [25, 26]; this study showed a trend towards improved local control with SRS, but did not attain statistical significance possibly due to the short radiographic follow-up periods and the small patient cohort.

Radiosensitizers are being studied for brain metastases for a variety of tumor types to improve intracranial disease control rates. Gadolinium-based nanoparticles and BRAF inhibitors concurrent with RT are being explored for melanoma brain metastases [27, 28]. Small tyrosine kinase inhibitors, small molecule HER2 targeting agents, temozolomide, and topotecan are being investigated with breast cancer [29, 30]. Furthermore in non-small cell lung cancer brain metastases, cisplatin and pemetrexed with concurrent cranial RT is actively being investigated [31]. For metastatic GI malignancies, there has been promising research done into radiosensitization with irinotecan, oxaliplatin, and biological agents for non-cranial sites [32–34]. Drawing upon this research, there is potential for concurrent novel therapies to improve the efficacy of cranial irradiation [32–35]. While dose escalation has been shown to improve tumor control in radioresistant histologies, large tumor size would limit this strategy for many cases [15, 36].

The role of WBRT and SRS in the management of brain metastases is evolving in the era of new systemic therapies [37]. In our study, overall survival was not significantly different between patients who received upfront SRS versus WBRT, however we did observe higher distant brain failure rates with SRS alone. Additionally, the association with ECD suggests tumor intracranial seeding as an important factor. A study by Aoyama et al. found no improvement in survival for the use of WBRT plus SRS, but did find the rate of intracranial relapse to be increased in those who received SRS alone similar to our series [25]. Sneed et al. also found no difference in survival when comparing SRS vs. WBRT and SRS [23]. A study by Chang et al. demonstrated an increased decline in learning and memory function in patients treated with SRS and WBRT vs. SRS, and an improved 4-month OS in the SRS alone group; however, the local and distant control was improved in the SRS plus WBRT group [14]. It is important to note that these studies were analyzing brain metastasis in general and not brain metastases from GI primaries as in the present study. Current research is exploring the utilization of SRS for greater than five brain metastases where historically WBRT was the preferred treatment modality. Yamamoto et al. demonstrated promising results when comparing patients with two to four brain metastases versus five to ten brain metastases treated with SRS and found non-inferiority in overall survival [15]. Compared to WBRT, SRS is associated with a shorter treatment duration, higher BED, and reduced neurocognitive deficits [14, 15].

Limitations of this study include its retrospective nature and the small sample size due to the relative rarity of this patient population. Additionally, given the short radiographic follow-up and the inclusion of cavitary lesions, it is often challenging to differentiate between radionecrosis and progression. Nevertheless, due to the relatively poor prognosis associated with brain metastases from GI primaries, future research should continue to assess prognostic and predictive factors, optimal dose fractionation schedules, treatment paradigms, and adverse effects. Future research should also attempt to discern the effect of radiation therapy on patient reported outcomes and quality of life endpoints. As survival improves for metastatic GI cancer with advances in systemic therapies, the incidence of brain metastasis will likely rise necessitating further improvement in treatments.

## Conclusion

Survival and intracranial disease control are poor following RT for brain metastases from GI primaries. In this small series, outcomes are worse than published series for other primary malignancies metastatic to the brain and further research into methods of local control improvement is warranted. The poor overall survival and local control could potentially be due to (1) radioresistant biology of GI malignancies, and (2) brain metastases in GI malignancies are generally a late event with the majority of patients presenting with diffuse active extracranial disease. The association found between extracranial disease and the development of new intracranial disease should be further explored. Future studies should elucidate the utility of dose escalation and/or radiosensitization in this patient population.

## Abbreviations

BED: Biologically equivalent dose
CI: Confidence interval
CT: Computed tomography
ECOG: Eastern Cooperative Oncology Group
GI: Gastrointestinal
GKS: Gamma Knife Surgery
GTV: Gross Tumor Volume
HR: Hazard ratio
KPS: Karnofsky Performance Status
LINAC: Linear accelerator
MR: Magnetic resonance
MRI: Magnetic resonance imaging
MVA: Multivariate analysis
N: Number
PET: Positron emission tomography
RT: Radiation therapy
SRS: Stereotactic radiosurgery
UVA: Univariate analysis
WBRT: Whole brain radiation therapy
